# Redox Control of the Immune Response in the Hepatic Progenitor Cell Niche

**DOI:** 10.3389/fcell.2020.00295

**Published:** 2020-05-06

**Authors:** Francesco Bellanti, Giuseppe Pannone, Nicola Tartaglia, Gaetano Serviddio

**Affiliations:** ^1^Center for Experimental and Regenerative Medicine, Institute of Internal Medicine, Department of Medical and Surgical Sciences, University of Foggia, Foggia, Italy; ^2^Institute of Anatomical Pathology, Department of Clinical and Experimental Medicine, University of Foggia, Foggia, Italy; ^3^Institute of General Surgery, Department of Medical and Surgical Sciences, University of Foggia, Foggia, Italy

**Keywords:** hepatic progenitor cell niche, redox balance, immune response, liver regeneration, reactive species

## Abstract

The liver commonly self-regenerates by a proliferation of mature cell types. Nevertheless, in case of severe or protracted damage, the organ renewal is mediated by the hepatic progenitor cells (HPCs), adult progenitors capable of differentiating toward the biliary and the hepatocyte lineages. This regeneration process is determined by the formation of a stereotypical niche surrounding the emerging progenitors. The organization of the HPC niche microenvironment is crucial to drive biliary or hepatocyte regeneration. Furthermore, this is the site of a complex immunological activity mediated by several immune and non-immune cells. Indeed, several cytokines produced by monocytes, macrophages and T-lymphocytes may promote the activation of HPCs in the niche. On the other side, HPCs may produce pro-inflammatory cytokines induced by liver inflammation. The inflamed liver is characterized by high generation of reactive oxygen and nitrogen species, which in turn lead to the oxidation of macromolecules and the alteration of signaling pathways. Reactive species and redox signaling are involved in both the immunological and the adult stem cell regeneration processes. It is then conceivable that redox balance may finely regulate the immune response in the HPC niche, modulating the regeneration process and the immune activity of HPCs. In this perspective article, we summarize the current knowledge on the role of reactive species in the regulation of hepatic immunity, suggesting future research directions for the study of redox signaling on the immunomodulatory properties of HPCs.

## Introduction

The liver is provided of exclusive regenerative capacity after consistent damage of various origin (viral, toxic, metabolic, genetic, or immunologic). Hepatocyte loss is replaced by the remaining functional parenchymal cells in the healthy liver (Michalopoulos, 2013). Nevertheless, a persistent or severe liver damage overwhelms the replication capacity of adult hepatocytes, and injured cells are replaced by the activation/replication of hepatic progenitor cells (HPCs) (Espanol-Suner et al., 2012). HPCs are characterized by an oval-shaped nucleus and a high nucleus-cytoplasm ratio, and express markers of both hepatocyte and biliary lineages (Thorgeirsson, 1996). However, the precise characterization of HPCs is a major challenge: even though several markers are now identified and employed, many are not specific for HPCs. Indeed, single markers are not able to accurately identify HPCs, as most of these molecules are either expressed by other hepatic cell types or upregulated upon inflammation (Lukacs-Kornek and Lammert, 2017). Nevertheless, the simultaneous expression of biliary cytokeratins (e.g., CK7/19) and conventional stem cell markers (e.g., Sox9, CD44, CD133, Epithelial Cell Adhesion Molecule—EpCAM, and Neural Cell Adhesion Molecule—NCAM) may allow HPC unique identification (Overi et al., 2020).

**FIGURE 1:**
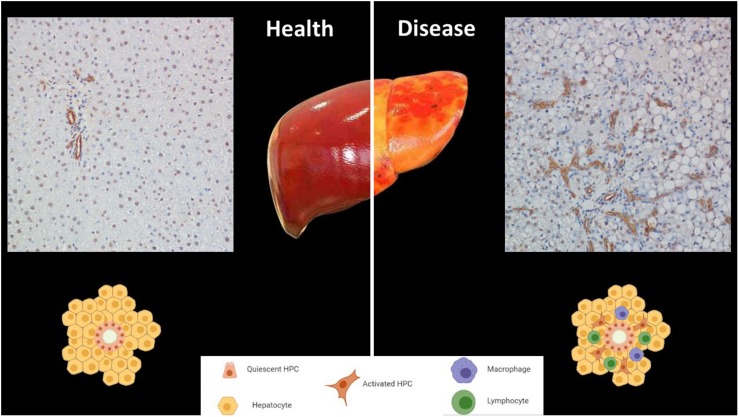
The niche of hepatic progenitor cells (HPCs) in health and disease. HPCs are in the most peripheral and smallest branches of the biliary tree, (canals of Hering). HPCs can be identified by immunohistochemistry through their cytokeratin19-positivity (brown). The top left panel shows a normal portal tract in a healthy human liver. The top right panel is representative of ductular reactions in a sample of a patient affected by non-alcoholic steatohepatitis, in which HPC expansion occurs (Magnification 200x). The bottom panel displays simplified drawings of the niche in both conditions.

HPCs are found in niches located within the smallest branches of the biliary tree, named Canals of Hering, at the interface between the hepatic parenchyma and the portal tract (Itoh and Miyajima, 2014). Further hepatic sites can transiently provide a niche for HPCs, such as the space of Disse and the central vein (Chen et al., 2017). Nevertheless, the HPC niche is defined not only by the site where it is located, but also by the composition of the niche. The HPC niche is a special microenvironment composed by different cell types and a scaffold of extracellular matrix, in which cytokines and growth factors released by the niche cells modulate signaling pathways for the regulation of H self-maintenance, proliferation, activation, transition, and differentiation (Theise, 2006). HPCs in the niche are found in association with other progenitors, such as angioblasts, precursors to hepatic stellate cells and endothelial cells (Carpino et al., 2016). These progenitors contribute to the stemness of the niche by releasing paracrine signals, which include matrix factors (hyaluronans, types III and IV collagens), minimally sulfated proteoglycans, and laminins and soluble signals such as leukemia inhibitory factor (LIF), hepatocyte growth factor (HGF), stromal derived growth factor (SDGF), and epidermal growth factor (EGF) (Carpino et al., 2016). In a quiescent state, the niche microenvironment maintains the progenitor phenotype and inhibits cell differentiation. Several types of both acute injury and chronic liver diseases give rise to the “ductular reaction”, in which the perturbation of the niche microenvironment starts the differentiation of HPCs toward a hepatocyte or cholangiocyte phenotype (Figure 1). The mechanisms by which HPCs acquire divergent cell fates in the adult liver rely on how the niche microenvironment is modulated to achieve a defined progenitor specification (Boulter et al., 2013).

Since HPC activation is the first step in progenitor-dependent regeneration, a complete knowledge of the mechanisms by which these cells are activated to proliferate and differentiate is important for the development of new therapies for liver disease. The niche is the site of a complex immunological activity mediated by several immune and non-immune cells. Indeed, several cytokines produced by monocytes, macrophages and T-lymphocytes may promote the activation of HPCs in the niche. On the other side, HPCs may be a source of cytokines and chemokines induced by liver inflammation. Several factors released by immune cells which mediate the HPC response to liver damage were described (Akhurst et al., 2005; Knight et al., 2005, 2007; Nguyen et al., 2007). Within the niche, a key signaling to control HPCs activation and proliferation is triggered by macrophages via the tumor-necrosis-factor-like weak inducer of apoptosis (TWEAK) (Bird et al., 2013). A further immune-mediated signaling upregulated in human chronic liver diseases consists in the chemokine stromal cell-derived factor 1 (SDF-1) and its receptor (CXCR4): SDF-1 is produced by HPCs and attracts CXCR4-positive inflammatory cells (Hatch et al., 2002; Hao et al., 2015).

Chronic liver diseases are characterized by a disruption of redox balance caused by an excess of reactive species (Jadeja et al., 2017). Reactive species modulate and are modulated by several transcription factors, which may be directly or indirectly involved in the regulation of stem/progenitor cell fate (di et al., 2018). Even though a direct regulation of HPCs by redox signaling has not been yet demonstrated, the main redox-dependent transcription factors are emerging as determinant and physiological modulators of stem and progenitor cells. The Hypoxia Inducible Factor 1α (HIF-1α) is one of the well-defined redox-dependent modulators of stem cell fate. Since regenerative niches are characterized by a hypoxic environment, cells stabilize HIF-1α which can modulate specific effectors, such as Notch, Wnt and Oct4 that control proliferation, differentiation and pluripotency (Mazumdar et al., 2009). Another stress-responsive transcription factor, the Nuclear factor erythroid 2-related factor 2 (NRF2), is a pivotal regulator of both pluripotent and adult stem cell biology in response to various environmental signals (Dai et al., 2020). The forkhead box protein (FoxO) family, involved in the resistance to oxidative stress by upregulating antioxidant enzymes, is implicated in the regulation of stem cell maintenance and integrity (Murtaza et al., 2017). Furthermore, cellular actions of reactive species include promotion or suppression of inflammation and immunity (Nathan and Cunningham-Bussel, 2013; Franchina et al., 2018). Consequently, the overwhelming production of reactive species could impact the secretory pattern of the immune and non-immune cells within the niche. Nevertheless, this aspect needs to be deepened and broadened by future investigations.

After a basic introduction on redox biology, the present perspective article will focus on redox-dependent pathways involved in immune regulation in liver diseases, providing support for redox signaling as a key factor in the immune-mediated processes of HPCs in the niche.

## Redox Biology and Oxidative Stress in Liver Diseases

Reactive species are highly unstable compounds classified as free radicals (characterized by one or more unpaired electrons in their outer shell) and non-radical derivatives (Table 1). These include reactive oxygen (ROS) and e nitrogen species (RNS), also named oxidants (Sies et al., 2017). In particular, ROS are mostly (but not exclusively) generated in mitochondria by the electron transport chain (Sohal et al., 1990; St-Pierre et al., 2002), and maintained at low concentrations by both the cytosolic and the mitochondrial antioxidant system, represented by enzymes such as superoxide dismutase, catalase, peroxidases, and non-enzymatic scavengers such as reduced glutathione (Mates and Sanchez-Jimenez, 1999). Reactive species act as second messengers at low concentrations, being involved in different processes such as apoptosis, cell proliferation, metabolism, and immunity (Laloi et al., 2004; Serviddio et al., 2013a; Holmstrom and Finkel, 2014; Liang and Ghaffari, 2014). On the contrary, exceeding oxidants induce oxidative stress, with damage to biological macromolecules, impaired cellular function and compromised cell viability (Mates and Sanchez-Jimenez, 1999; Ott et al., 2007; Bigarella et al., 2014).

**TABLE 1:** Metabolic reactions give rise to reactive oxygen and nitrogen species (ROS and RNS, respectively), generally named oxidants.

**Reactive Oxygen Species (ROS)**		**Reactive Nitrogen Species (RNS)**	
**Radicals**	**Non-Radicals**	**Radicals**	**Non-Radicals**
**O_2_^–^** ∙ Superoxide	**H_2_O_2_** Hydrogen peroxide	**NO** ∙ Nitric Oxide	**ONOO^–^** Peroxynitrite
**HO** ∙ Hydroxyl	**^1^O_2_** Singlet oxygen	**NO_2_** ∙ Nitrogen dioxide	**ROONO** Alkyl peroxynitrites
**HOO** ∙ Hydroperoxyl	**O_3_** Ozone		**N_2_O_3_** Dinitrogen trioxide
**L** ∙ Lipid radical	**LOOH** Lipid hydroperoxide		**N_2_O_4_** Dinitrogen tetroxide
**LOO** ∙ Lipid peroxyl	**HOCl** Hypochlorite		**HNO_2_** Nitrous acid
**ROO** ∙ Peroxyl			**NO_2_^+^** Nitronium anion
**LO** ∙ Lipid alkoxyl			**NO^–^** Nitroxyl anion
**RS** ∙ Thiyl radical			**NO^+^** Nitrosyl cation
**P** ∙ Protein radical			**NO_2_Cl** Nitryl chloride

*These reactive species can be classified as free radicals, characterized by one or more unpaired electrons in their outer shell, and non-radical derivatives.*

Characterized by intense metabolic activity, the liver is determinant for the overall redox state of the organisms. Several enzymes may produce reactive species in the liver, such as diamine oxidase, dehydrogenases, and the cytochrome P450 system. In particular, mitochondria and cytochrome P450 enzymes in hepatocytes, Kupffer cells, and neutrophils are the main sources of oxidants. Acute and chronic liver diseases are characterized by high production of reactive species (Roskams et al., 2003), which disrupt metabolism and cell cycle in hepatocytes, activate Kupffer cells, trigger collagen production by stellate cells and angiogenesis (Muriel, 2009).

The progression of liver damage during chronic viral hepatitis is also determined by oxidative stress. Hepatitis B virus (HBV) proteins trigger the production of reactive species: HBV X protein (HBx) localizes within mitochondria and alters transmembrane potential with consequent generation of reactive species (Rahmani et al., 2000; Henkler et al., 2001; Waris et al., 2001), which in turn activate signaling pathways promoting hepatocellular transformation (Chen and Siddiqui, 2007). Oxidative damage in hepatitis C infection is caused by chronic inflammation, iron overload, but also directly by hepatitis C virus (HCV) proteins (Choi and Ou, 2006). Indeed, the non-structural protein 5 A disrupts intracellular Ca^2+^ signaling, triggering mitochondrial production of radical species (Gong et al., 2004). Furthermore, the HCV core protein may cause oxidative damage with a direct effect on mitochondria (Okuda et al., 2002), and HCV proteins disrupt mitochondrial calcium homeostasis, leading to both bioenergetic impairment and nitro-oxidative stress (Piccoli et al., 2009). Increased reactive species production is also extensively described in acute or chronic alcohol consumption and related to the oxidant properties of ethanol (Cederbaum et al., 2009). Alcohol is mainly oxidized into acetaldehyde in a NAD^+^-dependent process catalyzed by alcohol dehydrogenase and by the microsomal ethanol oxidation system, based on cytochromes P450. After oxidation, most acetaldehyde is converted into acetate by cytosolic and mitochondrial aldehyde dehydrogenase in another NAD^+^-dependent process. Ethanol oxidation leads to ROS production, mainly hydrogen peroxide and superoxide anion (Cederbaum et al., 2009). Since these ROS are characterized by high reactivity and short half-life, they quickly bind to ethanol or iron atoms to form hydroxyl radical, ferrous oxide or hydroxyethyl radical, accounting for lipid peroxidation of cell membranes. Mitochondria (through the respiratory chain), the endoplasmic reticulum (through cytochrome P450) and Kupffer cells (through NADPH oxidase) are the main sources of ROS (Louvet and Mathurin, 2015). An impairment in redox balance is also described in non-alcoholic fatty liver disease (NAFLD), where free fatty acid excess causes overproduction of reactive species mostly by mitochondria and cytochrome P450 (Serviddio et al., 2011a, b, 2013b; Bellanti et al., 2017, 2018), which lead to a pro-oxidative environment triggering the release of pro-inflammatory cytokines, which in turn activate hepatic stellate cells to produce connective tissue; moreover, oxidative stress stimulates Kupffer cells and leads to hepatocellular apoptosis mediated by the expression of death receptor Fas-ligand (Koek et al., 2011). Reactive species are the most relevant cause of cellular disruption in hepatic ischemia/reperfusion (Bellanti, 2016). Oxidant injury is triggered by Kupffer cells and further amplified by polymorphonuclear leukocytes (Jaeschke and Farhood, 2002). Oxidative stress is involved in autoimmune hepatitis, providing a mechanism which links hepatic necroinflammation to fibrogenesis and disease progression (Pemberton et al., 2004). Catalase is one of the autoantigens in primary sclerosing cholangitis, suggesting that a redox unbalance caused by catalase antibodies could contribute to its pathogenesis (Orth et al., 1998). Oxidative stress is also a determinant for liver damage during cholestasis (Arduini et al., 2011), in which it is particularly marked in liver mitochondria (Serviddio et al., 2004). The molecular damage induced by oxidants is also a common pathway to several toxic agents and may lead to drug-induced liver injury, since this organ is the primary entrance for ingested drugs and is provided of several metabolizing enzymes (Krahenbuhl, 2001). A classic example of liver redox balance impairment by a toxic compound is provided by acetaminophen, whose hepatic metabolism is mediated by UDP-glucuronosyltransferases, sulfotransferases, and other cytochrome P450 enzymes which produce a reactive intermediate that can bind to sulfhydryl groups, deplete liver glutathione (GSH), and modify cellular proteins, leading to oxidative stress and mitochondrial damage (McGill and Jaeschke, 2013). Chronic inflammation associated with severe oxidative stress mediates hepatocarcinogenesis (Seitz and Stickel, 2006). The extent of oxidants is increased in hepatocellular carcinoma tissue rather than in non-cancerous liver (Iwagaki et al., 1995). Oxidative stress may boost the malignancy of hepatocellular carcinoma by triggering telomerases and angiogenesis (Nishikawa et al., 2009; Jo et al., 2011).

## Redox Modulation of the Hepatic Immune Response

Oxidative changes are determinant for the immune response and inflammation, which is induced by tissue damage and infection, favoring the removal of damaged/foreign components and resulting in tissue repair. The immune response can be classified into innate and acquired. Innate immunity is a primitive response characterized by lack of specificity, where the main actors are macrophages, neutrophils and dendritic cells, which recognize foreign bodies through the toll-like receptors (TLR) and activate other cells by secreting several cytokines or by presenting antigens on their membrane surfaces (Takeda, 2005). Even though innate immunity is generally considered non-specific and characterized by basic mechanisms, there is consistent evidence to support a high degree of cell type and stimulus specificity in its responses, also showing aspects of immunological memory (Smale et al., 2014; Sun et al., 2014). On the other side, adaptive immunity is highly specific and systematically organized, providing enduring protection with immunological memory (Shuai et al., 2016). Cells of the innate immune response may produce high levels of reactive species, leading to tissue damage and inflammation (Matsuzawa et al., 2005). Other than the oxidative burst, redox balance plays an important role in both the innate and the adaptive immune response, taking part to the macrophage, lymphocyte and dendritic cell signaling, or modulating the cytokine production (Yang et al., 2013).

The regulatory effect of oxidants on immunity was first evidenced when hydrogen peroxide at micromolar concentrations was able to activate the transcription factor nuclear factor-κB (NF-κB), a key determinant of the immune response; this effect was largely missing after a co-treatment with the antioxidant N-acetylcysteine (Schreck et al., 1991). Since then, several investigations addressed the immune-regulatory properties of oxidants in mild concentration, but also of oxidoreductant enzymes (Mullen et al., 2019).

Impairment of redox balance and the immune response are tightly interconnected in several liver diseases (Table 2) (Li et al., 2016). In alcoholic liver disease, chronic ethanol-induced production of reactive species dysregulates the production of cytokines as well as the signaling mediated by the Toll Like Receptor-4 in Kupffer cells (Cohen et al., 2011). Alcoholic steatohepatitis (ASH) is also determined by the activation of Notch1-NF-κB signaling pathway induced by redox disbalance in hepatocytes (Wang et al., 2014). A further study suggested that ethanol exposure triggers a distinct cytokine secretory pattern in Kupffer cells or hepatocytes, since oxidants differentially modulate the production of pro-inflammatory cytokines via NF-κB signaling, mRNA stability, and histone acetylation (Dong et al., 2016). Other than ASH, the link between redox alterations and immune response during non-alcoholic steatohepatitis (NASH) is provided by several pieces of evidence. Despite a substantial literature supporting the importance of innate immunity, NASH is characterized by the presence of immunoglobulins against oxidation products, able to recruit B- and T-lymphocytes which in turn amplify the activation of macrophages and natural killer (NK) cells, leading to liver fibrosis (Sutti and Albano, 2019). Furthermore, reactive species negatively modulate the immune suppression, promoting the apoptosis of hepatic regulatory T cells and inhibiting the expansion of hepatic myeloid-derived suppressor cells (Ma et al., 2007; Resheq et al., 2015). The immune response triggered by redox imbalance is typically involved in hepatic ischemia-reperfusion injury (Prieto and Monsalve, 2017). Indeed, overproduction of reactive species following reperfusion acutely activates Kupffer cells with consequent release of pro-inflammatory cytokines (Jaeschke and Ramachandran, 2011). Next, elevated oxidant levels amplify the inflammatory cascade through the direct activation of NF-κB, but also increasing the levels of tumor growth factor-β (TGF-β), tumor necrosis factor (TNF), and interleukin-1β (Mukhopadhyay et al., 2012). The involvement of redox signaling in immune response is also extensively demonstrated in idiosyncratic drug-induced liver injury, the unpredictable hepatic reaction to drugs (Mak and Uetrecht, 2017). Through redox alterations, several drugs are able to expose hepatocytes to cytokines, such as TNF (Han et al., 2009). For instance, toxicants such as acetaminophen or chlorpromazine disrupt redox balance with consequent activation of c-Jun N-terminal kinase (JNK) and/or inhibition of NF-κB activity, both important in sensitization to TNF (Gandhi et al., 2010).

**TABLE 2:** A comprehensive but not exhaustive overview on the current knowledge about the main immunologic pathways and redox-dependent mechanisms involved in the pathogenesis of liver diseases.

**Liver disease**	**Immune response**	**Redox involvement**
Hepatitis B	Innate immunity is triggered by viral nucleic acids and proteins. Recruitment of the adaptive immune system, functional development and expansion of distinctive B- and T-cell clones. Generation of a memory response (Tan et al., 2015).	Reactive species promote Raf-1 translocation within mitochondria, contributing to the onset of hepatocellular carcinoma (Chen and Siddiqui, 2007).
Hepatitis C	HCV replication triggers pathogen-associated molecular patterns (PAMPs), which in turn stimulate IFN and the expression of IFN-stimulated genes (ISGs). IFNs, ISGs, cytokines, and other signals resulting from infected hepatocytes sustain the initiation and modulation of the adaptive immune response (Dustin, 2017).	Overproduction of reactive species induced by HCV proteins leads to intrahepatocellular events, promoting the progression of hepatic and extrahepatic complications of HCV infection (Choi and Ou, 2006).
Alcoholic hepatitis	Early stage: impaired barrier function of the intestinal mucosa leads to increased lipopolysaccharide (LPS) to the portal circulation, with further activation of innate immunity via Toll-like receptors (TLRs). Late stage: alcohol oxidative products inhibit natural killer (NK) cells that induce apoptosis of activated hepatic stellate cells (HSCs) to limit fibrosis. Cytokines produced by inflammatory macrophages and Kupffer cells activate quiescent HSCs, leading to the proliferation of myofibroblasts that produce extracellular matrix proteins. CD8+ T-lymphocytes further contribute to HSCs activation (Li et al., 2019).	Reactive species produced by ethanol metabolism act directly on the transcriptional network that modulates both lipid metabolism and fibrogenesis, also promoting mutagenesis (Ceni et al., 2014).
Non-alcoholic fatty liver disease	Innate immunity: activation of the Nod-like receptor protein 3 (NLRP3) inflammasome components and Toll-like receptors (TLRs) by several ligands. Adaptive immunity: activation of Kupffer cells and release of proinflammatory cytokines, leading to the recruitment of bone marrow-derived monocytes and neutrophils that further contribute to the inflammatory response. B cells, Th1- and Th17-derived cytokines worsen the hepatic damage (Parthasarathy et al., 2020).	Reactive species promote the release of pro-inflammatory cytokines, which in turn activate hepatic stellate cells to produce connective tissue; moreover, reactive species activate Kupffer cells and lead to hepatocellular apoptosis mediated by the expression of death receptor Fas-ligand (Koek et al., 2011).
Autoimmune hepatitis	The inflammation seems to be secondary to both cell-mediated (T-cell) and humoral (B-cell) activity. A molecular mimicry activates an immune response directed towards self-proteins structurally like foreign pathogens. These proteins activate T cells which initiate and perpetuate liver injury (Kerkar and Chan, 2018).	Reactive species production is associated with T-cell activation and proliferation, but continued oxidants exposure induces T-cell hyporesponsiveness (Lee et al., 2016).
Cholestasis	Cholestatic hepatocytes start inflammatory response by cytokine release, with consequent neutrophil chemotaxis. IL-17 released by neutrophils and Th-cells stimulates Kupffer cells to produce proinflammatory and fibrogenic cytokines (Li et al., 2017).	Reactive species are mainly produced by bile acid toxic induction in hepatocytes, and by neutrophils. Oxidants promote proliferation, migration and collagen production by hepatic stellate cells (Copple et al., 2010).

## Redox Biology and Immune Regulation of Hepatic Progenitor Cells

In recent times, more than 170 clinical trials on stem cell-based therapies in liver diseases have been registered. Most of them are testing umbilical cord or bone marrow-derived mesenchymal stem cells, but their transplantation efficiency, clinical reliability, and long-term safety are very concerned (AdiwinataPawitan, 2019). HPCs are considered an alternative source for cell-based therapy, but their survival is low in injured liver (Chen et al., 2020). The key to use HPCs for transplantation relies on the effective and stead induction to differentiate into and exert the function of mature hepatocytes. It is then compulsory to define the molecular mechanisms involved in the modulation of HPCs biology, to develop standardized methods for successful human transplantation.

**FIGURE 2:**
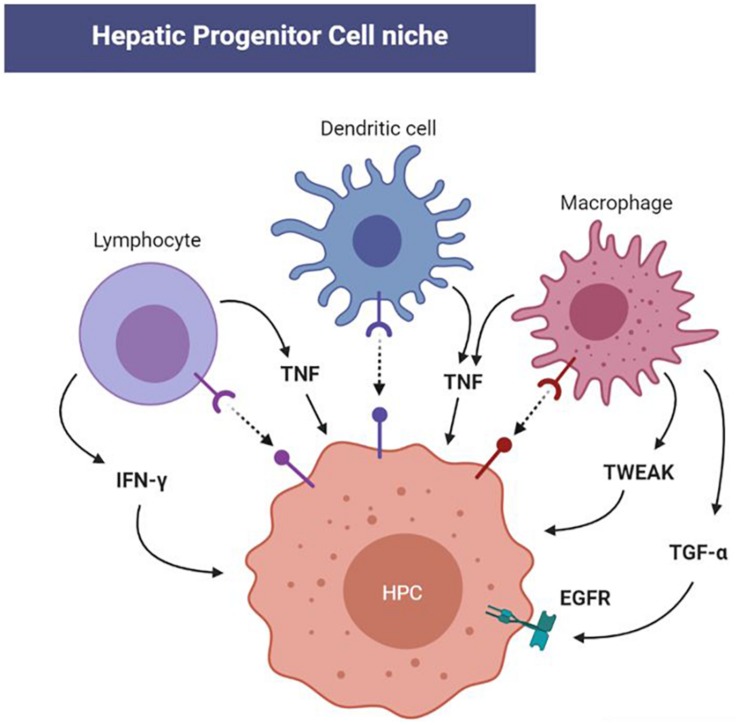
Redox-modulated cellular and molecular immune-mediators of hepatic progenitor cell (HPC) activation in the niche. The immune response triggered by both acute and chronic liver injury is outlined by the involvement of Kupffer cells, dendritic cells, and lymphocytes in the HPC niche. Progenitor activation is then initiated by several cytokines including tumor necrosis factor (TNF), TNF-like weak inducer of apoptosis (TWEAK), interferon-γ, while epidermal growth factor receptor (EGFR) can be activated by transforming growth factor-α (TGF-α). Since these cytokines and corresponding pathways can be redox-modulated, future investigations will clarify the impact of redox alteration in HPC homeostasis through the immune response.

The immune system is becoming evident as a determinant modulator of HPC niches in the adult liver, since several cytokines and chemokines regulate stemness, proliferation, activation, and cell fate of HPCs in physiological and pathological conditions. HPC proliferation occurs with an intrahepatic immune response, characterized by the recruitment of Kupffer cells, dendritic cells, and lymphocytes accounting for local cytokine secretion with mitogen properties (Figure 2), which stimulate HPC expansion (Strick-Marchand et al., 2008). Indeed, early activation of HPCs is triggered by several mediators of the hepatic immune response including TNF, TWEAK, interferon-γ (IFN-γ), interleukin-6, leukemia inhibitory factor, epidermal growth factor (EGF), transforming growth factor-α (TGF-α), and lymphotoxin-β (Lukacs-Kornek and Lammert, 2017). Most of these cytokines and growth factors are modulated by reactive species and redox signaling. The following observations, mostly coming from gene modified mice and models, are strongly evocative of the involvement of redox alterations in the immune-mediate regulation of HPC quiescence/activation.

TNF-induced HPC activation may be an important component of inflammatory injury in the liver, particularly in chronic inflammation, when TNF and reactive species are secreted simultaneously. Indeed, TNF receptor type 1 knockout mice show impairment of HPC proliferation, and a reduction of TNF-mediated cytosolic oxidant production (Knight et al., 2000; Hardin et al., 2008). In this scenario, redox changes induced by oxidants would expose hepatocytes to the lethal actions of TNF, promoting regeneration via HPCs.

∙ TWEAK induces multiple pathways of cell death via reactive species (Nakayama et al., 2003), indicating that redox signaling could be involved in TWEAK-mediated hepatocellular necrosis, and at the same time suggesting the activation of HPCs. In fact, mice lacking TWEAK receptor (Fn14) do not show any HPC proliferation (Jakubowski et al., 2005), and genetic silencing of Fn14 stops the TWEAK-induced production of reactive species in macrophages (Madrigal-Matute et al., 2015).

∙ Oxidants exert an inhibitory effect on the production of IFN-γ by T cells (Abimannan et al., 2016), nevertheless hepatic NK cells are resistant to this inhibitory effect (Zhang et al., 2003). On the other side, IFN-γ receptor knockout mice exhibit a higher rate of reactive species production (Espejo et al., 2002). Since during T cell-mediated hepatitis, the alteration of IFN-γ secretion impairs hepatocellular regeneration and promotes NK cell-sensitive HPC expansion (Hines et al., 2007), this process could be redox-modulated.

∙ The EGF receptor (EGFR) signaling pathways has been demonstrated to regulate the liver progenitor cell compartment (Komposch and Sibilia, 2015). EGFR ligands such as EGF and TGF-α are determinant in the maintenance of HPC phenotype, preventing the epithelial-mesenchymal transition which initiates tumor transformation (Wang et al., 2015). Loss of EGFR suppresses HPC differentiation toward cholangiocyte promoting hepatocyte differentiation (Kitade et al., 2013). Reactive species such as hydrogen peroxide function as second messengers in the modulation of EGFR signaling (Truong and Carroll, 2012), suggesting that this pathway could be redox regulated in HPCs during liver diseases.

Taken together, these data represent a solid background on which further specific investigations can be based on.

## Conclusion

In regenerative niches, low levels of oxidants contribute to the quiescence of stem cells, whereas high amounts of reactive species promote the activation and differentiation of progenitors. Redox alterations play a determinant role in both acute and chronic liver diseases, inhibiting the proliferation of hepatocytes and increasing the number of HPCs. However, the association mechanism between redox imbalance and HPC proliferation is not clearly established. Redox biology is more and more acknowledged as a main contributor to the immune response, nevertheless its role in immune-modulated HPC homeostasis needs to be extensively investigated.

Understanding the underlying molecular mechanisms involved in the redox regulation of immune function in the hepatic progenitor niche will provide significant new insights into the biology of HPCs and liver regeneration.

## Author Contributions

FB wrote the first draft of the manuscript. GP and GS wrote sections of the manuscript. NT substantially contributed to the conception of the new version of the work. All authors contributed to manuscript revision, read and approved the submitted version.

## Conflict of Interest

The authors declare that the research was conducted in the absence of any commercial or financial relationships that could be construed as a potential conflict of interest.
